# Role of PTEN, PI3K, and mTOR in Triple-Negative Breast Cancer

**DOI:** 10.3390/life11111247

**Published:** 2021-11-17

**Authors:** Mirjana Prvanović, Milica Nedeljković, Nasta Tanić, Tijana Tomić, Tanja Terzić, Zorka Milovanović, Zlatko Maksimović, Nikola Tanić

**Affiliations:** 1 Institute of Pathology, Faculty of Medicine, University of Belgrade, 11000 Belgrade, Serbia; mirjanaprvanovic@gmail.com (M.P.); tatjana.terzic63@gmail.com (T.T.); 2 Department of Experimental Oncology, Institute of Oncology and Radiology of Serbia, 11000 Belgrade, Serbia; 3 Department of Radiobiology and Molecular Genetics, Institute of Nuclear Sciences “Vinča”, National Institute of Republic of Serbia, University of Belgrade, 11000 Belgrade, Serbia; nastad@vinca.rs (N.T.); tijana.tomic@vin.bg.ac.rs (T.T.); 4 Department of Pathology, Institute of Oncology and Radiology of Serbia, 11000 Belgrade, Serbia; zmilovanovic@ncrc.ac.rs; 5 Public Health Institution Hospital “Sveti Vracevi”, 76300 Bijeljina, Republika Srpska, Bosnia and Herzegovina; zlatko.maksimovic@gmail.com; 6 Department of Neurobiology, Institute for Biological Research “Siniša Stanković”, National Institute of Republic of Serbia, University of Belgrade, 11000 Belgrade, Serbia; nikolata@ibiss.bg.ac.rs

**Keywords:** triple-negative breast cancer, PTEN, PI3K, mTOR, protein expression, gene deletions, multidrug resistance, ABCG2, ABCC1, ABCB1

## Abstract

Breast cancer is the most commonly occurring malignancy and the leading cause of cancer-related death in women. Triple-negative breast cancer (TNBC) is the most aggressive subtype and is associated with high recurrence rates, high incidence of distant metastases, and poor overall survival. The aim of this study was to investigate the PI3K/PTEN/Akt/mTOR pathway as one of the most frequently deregulated pathways in cancer. We aimed to explore the impact of PI3K and mTOR oncogenes as well as the PTEN tumor suppressor on TNBC clinical behavior, prognosis, and multidrug resistance (MDR), using immunohistochemistry and copy number analysis by quantitative real-time PCR. Our results revealed that loss of PTEN and high expression of PI3K and mTOR proteins are associated with poor outcome of TNBC patients. PTEN deletions appeared as a major cause of reduced or absent PTEN expression in TNBC. Importantly, homozygous deletions of PTEN (and not hemizygous deletions) are a potential molecular marker of metastasis formation and good predictors of TNBC outcome. In conclusion, we believe that concurrent examination of PTEN/PI3K/mTOR protein expression may be more useful in predicting TNBC clinical course than the analysis of single protein expression. Specifically, our results showed that PTEN-reduced/PI3K-high/mTOR-high expression constitutes a ‘high risk’ profile of TNBC.

## 1. Introduction

Breast cancer is the most commonly occurring malignancy in women and the leading cause of cancer-related death in women worldwide [1]. Triple-negative breast cancer (TNBC) represents approximately 20% of breast cancer cases and is characterized by the lack of expression of estrogen (ER), progesterone (PR), and HER-2 receptors [2]. TNBC is the most aggressive breast cancer subtype and is associated with high recurrence rates, high incidence of distant metastases, and poor overall survival [3,4]. Additionally, TNBCs have a very high mitotic activity, which suggests the upregulation of growth factor signaling pathways and downregulation of inhibitors [5]. TNBC does not respond to currently available targeted therapy and often becomes resistant to chemotherapy [6]. Therefore, clarifying mechanisms of multidrug resistance (MDR) in TNBC is a priority.

Numerous mechanisms lead to the development of resistance of neoplastic cells to cytotoxic drugs, including overexpression of efflux pumps, abnormal tumor microenvironment, and other cellular/physiological pathways. One of the most important is the overexpression of ABC superfamily transporters. P-gp (ABCB1), MRP1 (ABCC1), and BCRP (ABCG2) are three major ABC transporters that confer many of the structural diversities related to anticancer drug resistance [7]. We confirmed elevated expression of these transporters in TNBC patients [8]. Moreover, we showed that expression of ABCB1 may be useful as a marker of metastatic spread [8].

Recent studies indicated that the phosphoinositide-3 kinase (PI3K)/phosphatase and tensin homolog (PTEN)/AKT-/mammalian target of rapamycin (mTOR) signaling pathway was involved in the generation of MDR by promoting the expression of membrane transporters. Once activated, the PI3K/PTEN/AKT/mTOR (PPAM) pathway enhances drug efflux by efficiently expressing ABC transporters and reducing the response to chemotherapeutic drugs, which further causes MDR. This is one of the most frequently activated pathways in many types of cancers [9], including breast cancer. That makes it one of the most important reasons for intrinsic resistance. Consequently, the PPAM pathway has emerged as a novel target for overcoming drug resistance, as summarized in [10,11].

The PPAM pathway is a crucial transduction system and one of the main mechanisms by which cells control growth, survival, and proliferation [12]. PI3K is a central hub that transduces signals from various growth factors, leading to the phosphorylation of mTOR. Activated mTOR provides tumor cells with a significant survival and growth advantage, owing to enhanced protein synthesis, decreased autophagy, and apoptosis [13,14]. PI3K activity is counterbalanced by the tumor suppressor PTEN, which acts as the principal negative regulator of the pathway [12]. Aberrations of the PPAM pathway have been associated with tumorigenesis in multiple human malignancies, including breast cancer [15]. However, significant controversy still exists regarding the effects of the aberrant PPAM pathway on breast cancer clinical behavior, especially in the TNBC subtype [16,17,18,19].

We hypothesized that alterations of the PPAM pathway could be a driving force for TNBC’s aggressive nature and resistance to drugs. PI3K activation and PTEN downregulation are primary modes of PPAM pathway deregulation, while aberrations of mTOR itself are very infrequent [20]. This study aimed to explore the impact of PI3KT and mTOR oncogenes as well as the PTEN tumor suppressor on TNBC clinical behavior, prognosis, and MDR.

## 2. Materials and Methods

A total of 70 TNBCs diagnosed and surgically treated at the Institute for Oncology and Radiology of Serbia were investigated in this study. A cohort of 16 hormone receptor positive (HR+) breast cancers (about 20%) was included for control purposes. All examined tumor samples were formalin-fixed and paraffin-embedded. ER, PR, and HER2 statuses were routinely assessed before resectioning at the Department of Pathology, Institute for Oncology and Radiology of Serbia, using semi-quantitative, commercial IHC assay according to manufacturer recommendations. In short, ER and PR protein expressions were defined as negative if the Allred score was ≤3 [21], while HER2 expression was regarded as negative if the score was 0 or 1+ [22]. In the case of a 2+ score, HER2-negative status was confirmed by chromogenic in situ hybridization–CISH [22]. Detailed clinical records were available for included TNBC patients and are summarized in Table 1. All tumors were restaged based on the 2018th updated guidelines. The new staging system provides more accurate information and has higher prognostic power [23,24,25]. The mean follow-up period of patients was 104 months, with a range from 7 to 156 months. Approval for this study was obtained from the ethical committee of the Institute for Oncology and Radiology of Serbia, number 4321-01. All samples were collected and analyzed in accordance with the ethical standards laid down in the 1964 Declaration of Helsinki.

### 2.1. Immunohistochemistry

Archived tissue blocks were cut into 5 μm thick slices and placed onto SuperFrost plus slides (Thermo Scientific, Waltham, MA, USA). Following deparaffinization with xylene and rehydration with ethanol, epitope retrieval by heat was performed with the RE7116-CE epitope retrieval solution (Novocastra, Leica Biosystems, Germany). Endogenous peroxidase activity was blocked using 3% hydrogen peroxide, RE7101 (Novocastra, Leica Biosystems, Germany). Slides were then washed in tris-buffered saline and incubated with antibodies against PTEN, PI3K, and mTOR (1:100 dilution for each) for 60 min at room temperature. The following antibodies were used: polyclonal rabbit antibody 51-2400 (Invitrogen, Thermo Fisher Scientific, Waltham, MA, USA) for PTEN, monoclonal mouse antibody ab86714 (Abcam, Cambridge, UK) for PI3K, and polyclonal rabbit antibody ab25880 (Abcam, Cambridge, UK) for mTOR. Antibody-treated slices were stained with Novolink Polymer Detection System RE7140-K (Novocastra, Leica Biosystems, Germany) according to the manufacturer’s recommended protocol and contrasted with Mayer’s hematoxylin. Immunohistochemical analysis of ABC transporters ABCC1, ABCG2, and ABCB1 is described in [8].

### 2.2. Evaluation of Staining

**Figure 1:**
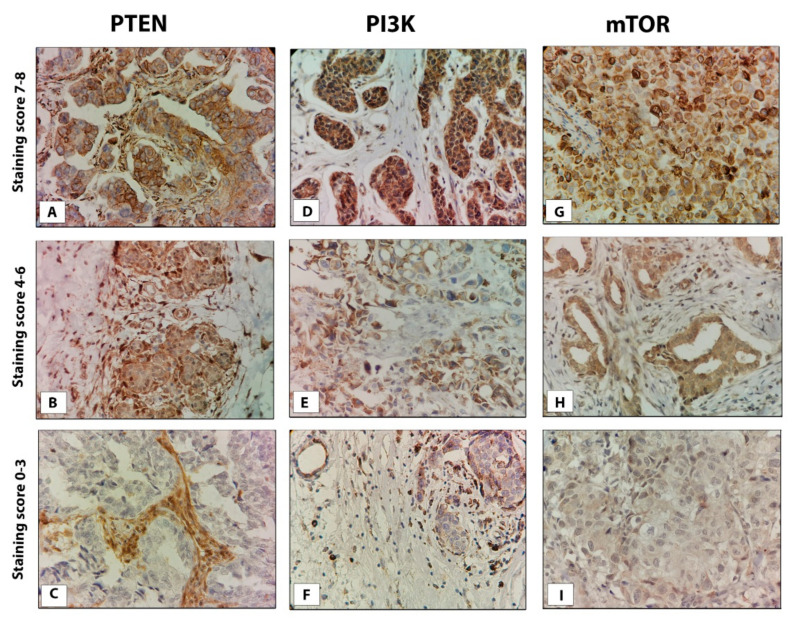
Representative images of PTEN, PI3K, and mTOR protein expression in TNBC tissue. (**A**) strong, (**B**) moderate, and (**C**) weak PTEN staining; (**D**) strong, (**E**) moderate, and (**F**) weak PI3K staining; (**G**) strong, (**H**) moderate, and (**I**) weak mTOR staining. Original magnification, ×40.

Staining was assessed independently by two pathologists blinded to clinical outcome, using a semi-quantitative method based on the: percentage of stained cells (0 = no immunoreactivity; 1 = <1%; 2 = 1–10%, 3 = 11–33%; 4 = 34–66%; 5 = 67–100%) and intensity of staining (0 = no immunoreactivity; 1 = weak; 2 = moderate; 3 = strong staining) (Figure 1). The sum of scores for the percentage of stained cells and the strength of their staining was used as a semi-quantitative measure of protein expression. Adjacent heathy tissue was used as an internal control.

The expression of PTEN was considered reduced or absent if the overall score was ≤3 and positive if the overall score was 4 or higher [26]. Since no standard cut-off values for the estimation of PI3K and mTOR immunohistochemical expressions have been defined to date, we used the median score as a cut-off point to designate low and high expression in TNBC. Accordingly, PI3K and mTOR statuses were categorized as follows: low expression if the score was ≤6 and high expression if the score was >6.

### 2.3. DNA Extraction

Genomic DNA was extracted from 70 TNBC samples using Kapa Biosystems Express Extract Kit (KK7151, Kapa Biosystems, Wilmington, MA, USA) according to the manufacturer’s recommendations. The quality of the isolated DNA was confirmed by agarose gel electrophoresis, while concentrations and purity were determined spectrophotometrically (NanoDrop Technologies, Wilmington, DE, USA). Isolated and purified DNA was stored at +4 °C.

### 2.4. PTEN Copy Number Analysis by Quantitative Real-Time PCR

Copy number of the *PTEN* gene was assessed using quantitative real-time PCR (qPCR) and Hs03007912_cn TaqMan assay (Applied Biosystems, Foster City, CA, USA). *RNase-P* was used as an internal control gene (4403326, Applied Biosystems). Calibration was done with DNA isolated from normal, paraffin-embedded breast tissue. All tumor samples were prepared in duplicate with normal controls being prepared in triplicate. The total reaction volume was 15 µL. For both *PTEN* and *RNase-P*, reactions contained 1× TaqMan Master Mix, 1× TaqMan Copy Number Assay, and 40 ng of DNA. Three normal DNA controls functioned as calibrators in each reaction. PCR amplification was carried out in the ABI Prism 7500 Sequence Detection System. Amplification protocol consisted of a denaturation phase at 95 °C for 10 min, followed by 40 amplification cycles at 95 °C for 15 s, and a step at 60 °C for 1 min. A mean Ct value of each duplicate was calculated and used for the relative quantitation of copy number according to the Livak 2-ΔΔCT method [27]. Each run also contained a no-template control. Generated results were analyzed by RQ Study Add ON software for 7500 v 1.3 SDS instrument with a confidence level of 95% (*p* < 0.05). Since all tumor samples invariably contain a portion of normal tissue, PTEN copy number was classified as hemizygous deletion if the average copy number ratio estimate given by qPCR was ≤0.8 and as a homozygous deletion if the ratio estimate was ≤0.4.

### 2.5. Statistical Analysis

Data analysis was performed using GraphPad Prism 8 software (GraphPad Software, Inc. San Diego, CA, USA) and IBM SPSS Statistics version 25 for Windows (IBM Corporation, Armonk, NY, USA). Correlations between the expressions of analyzed proteins (PPAM and ABC transporters) in TNBC were examined using Spearman’s rank test. The concordance of *PTEN* gene status and PTEN protein expression in TNBC was evaluated using Fisher’s exact test and a Kappa test. The comparison of PTEN/PI3K/mTOR protein expression level between triple-negative and hormone receptor-positive breast cancers was performed using the Mann–Whitney U test. Associations of clinicopathologic parameters of TNBC with the *PTEN* gene status and PTEN/PI3K/mTOR protein expression were determined using Fisher’s exact test, Fisher’s exact test with Freeman–Halton extension, or Chi-square test, depending on test conditions. Survival distributions were estimated by the Kaplan–Meier product-limit method, and the log-rank test was used to determine the significance of the differences between survival curves. Multivariate Cox regression was used to identify independent predictors of disease-free interval and overall survival. Disease-free interval (DFI) was calculated from the day after surgery to the first day of disease progression, while overall survival (OS) was calculated from the day after surgery to the last follow-up examination or death of the patient. All performed statistical tests were two-tailed. Statistical differences were considered significant for *p* ≤ 0.05.

## 3. Results

### 3.1. PTEN, PI3k, and mTOR Protein Expression

**Figure 2:**
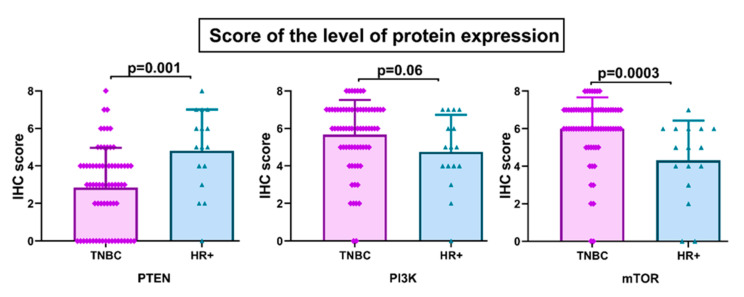
Level of PTEN, PI3K, and mTOR protein expression according to breast cancer subtype. IHC, Immunohistochemistry.

The levels and cellular distribution of protein expressions of PTEN, PI3K, and mTOR were analyzed by immunohistochemistry in 70 TNBC specimens. Positive PTEN immunoreactivity was observed in 43% of TNBC samples (30/70), while the remaining 57% (40/70) showed reduced or absent PTEN expression. The level of PTEN protein expression was significantly lower in TNBC compared to HR+ breast cancer (*p* = 0.001) (Figure 2). PTEN was predominantly expressed in the nucleus and cytoplasm. In case of PI3K and mTOR, 40% (28/70) and 44% (31/70) of TNBC specimens exhibited high protein expression, respectively. Low levels of PI3K and mTOR expression were detected in the remaining 60% and 56% of TNBC samples, respectively. In contrast to PTEN, the level of mTOR protein expression was significantly higher in TNBC compared to HR+ tumors (*p* = 0.0003) (Figure 2). Protein expression of PI3K was marginally higher in TNBC (*p* = 0.06) (Figure 2).

The analysis of the correlation between expressions of PI3K and mTOR in TNBC revealed significant concordance (np–70, Rho = 0.42, *p* = 0.0003). PI3K and mTOR were predominantly expressed in cytoplasm and plasma membrane.

### 3.2. Copy Number Alterations of PTEN Gene

Copy number status of the *PTEN* gene in TNBC was evaluated by qPCR in order to examine the genetic base of decreased PTEN protein expression. *PTEN* deletions were found in 44% (31/70) of samples. Hemizygous deletions were observed in 37% (26/70) of cases, while 7% (5/70) had homozygous deletions. The concordance rate of *PTEN* deletions with inactivation of protein expression was 87% (*p* < 0.0001, Kappa value 0.75) (Table 2). All tumor specimens with *PTEN* copy number aberrations (both hemi- and homozygous deletions) exhibited reduced protein expression. However, 13% (9/70) of samples with normal *PTEN* copy numbers had decreased protein expression.

### 3.3. Clinicopathologic Features According to PTEN, PI3K, and mTOR Protein Expression and PTEN Gene Copy Number

**Figure 3:**
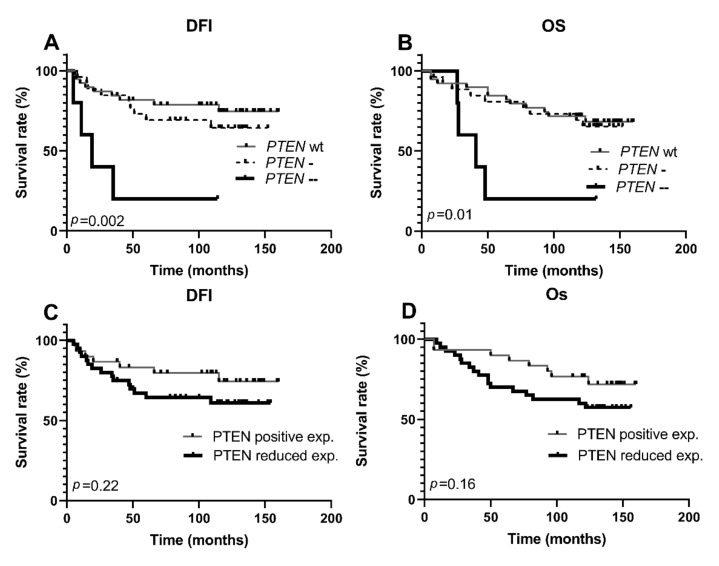
Kaplan–Meier survival curves according to PTEN copy number status and protein expression in the TNBC group. DFI, disease-free interval; OS, overall survival; Wt, wild-type; PTEN-, hemizygous deletion; PTEN--, homozygous deletion. (**A**) Patients with PTEN homozygous deletions had a significantly shorter DFI (**B**). Patients with PTEN homozygous deletions had a significantly shorter OS (**C**). Reduced PTEN expression did not affect patient disease-free interval (**D**). Reduced PTEN expression did not affect patient OS.

The relationship between PTEN expression/gene copy number aberrations and clinicopathological parameters of TNBC are summarized in Table 3. The tumors with complete loss of the *PTEN* gene were significantly more likely to metastasize to distant sites (*p* = 0.007), while no correlations were detected with hemizygous deletions. Following the same pattern, the inactivation of *PTEN* by homozygous deletions was significantly associated with shorter DFI (*p* = 0.002) and OS (*p* = 0.01) in univariate analysis (Figure 3).

Paralleling PTEN, PI3K, and mTOR expression profiles were also analyzed in relation to standard histopathological parameters of TNBC. High expression of PI3K was associated with lymph node metastases (*p* = 0.04), high pathological prognostic stage (*p* = 0.02), and tentatively associated with larger tumor size (*p* = 0.05). High expression of mTOR was also associated with a high pathological prognostic stage (*p* = 0.02). Data are summarized in Table 4.

**Figure 4:**
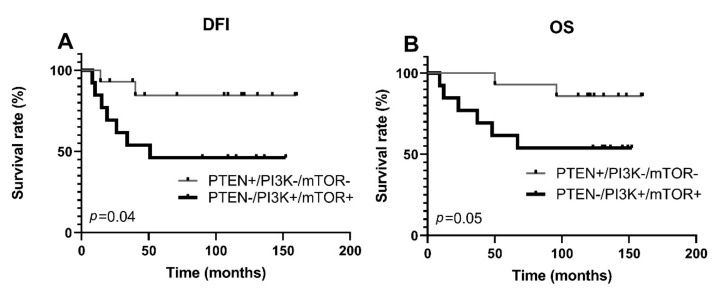
Kaplan–Meier survival curves according to PTEN, mTOR, and PI3K protein expressions in the TNBC group. DFI, disease-free interval; OS, overall survival. A. Patients with reduced PTEN and high mTOR/PI3K protein expression had a significantly shorter disease-free interval B. Patients with reduced PTEN and high mTOR/PI3K protein expression had a significantly shorter overall survival.

The expression levels of PI3K and mTOR did not affect TNBC outcome when both proteins were assessed independently from each other and PTEN expression (Supplementary Material, Figure S1). However, since the interplay between PTEN and PI3K influences mTOR activity, we next investigated whether simultaneously reduced PTEN expression and high PI3K/mTOR expressions could increase the risk of aggressive disease and poor outcome, while simultaneously positive PTEN expression and low PI3K/mTOR expressions could decrease it. Indeed, simultaneously reduced PTEN and high PI3K/mTOR expressions were associated with lymphatic invasion (*p* = 0.01), advanced tumor stage (*p* = 0.01), distant metastases (*p* = 0.03), shorter DFI (*p* = 0.04), and shorter OS (*p* = 0.05) in univariate analysis (Table 5, Figure 4).

To assess the independent prognostic effect of PTEN, PI3K, and mTOR protein expressions and *PTEN* gene copy number, we performed Cox multivariate regression analysis. The factors that significantly influenced TNBC, DFI, and OS in univariate analysis (Figure 3 and Figure 4, Supplementary Material, Table S1) were entered into the multivariate model. However, only distant metastasis status was shown to be an independent prognostic indicator of poor DFI with a HR (15.63, 95% CI 1.84–133.07, *p* = 0.01) and OS with a HR (18.68, 95% CI 5.65–61.73, *p* < 0.001) (Supplementary Material, Tables S2 and S3).

### 3.4. Activation of PI3K/PTEN/AKT/mTOR Pathway and Protein Expression of ABCG2, ABCC1, and ABCB1 Transporters

The expression of PTEN, PI3K, and mTOR was not correlated with the expression of ABCG2, ABCC1, and ABCB1 transporters in the entire TNBC cohort nor in the subgroups defined by clinicopathologic parameters: presence of lymphatic and distant metastasis (Supplementary Material, Table S4). The copy number status of the PTEN gene was not associated with the expression of the three examined ABC transporters in TNBC (data not shown).

## 4. Discussion

PTEN and PI3K are decisive controllers, while mTOR is the main effector, of a critical PI3K/PTEN/Akt/mTOR (PPAM) pathway that regulates cell proliferation, intrinsic drug resistance, and survival. Still, the potential role of over-activated PPAM in TNBC is controversial.

Our study showed that PTEN downregulation was significantly more prevalent in TNBC compared to HR+ disease, which implies that PTEN loss might promote the aggressive behavior of TNBC breast cancer. Detected high frequency of PTEN inactivation was in the range of some previous studies in which loss of PTEN expression varied from 44% [18] to 85% of TNBC cases [17,19].

In order to assess the genetic mechanisms responsible for the reduction of PTEN expression in TNBC, we analyzed *PTEN* gene copy number. Our investigation revealed that 37% of samples had hemizygous deletions, while 7% had homozygous deletions, a complete loss of the PTEN gene. The rate of *PTEN* copy number loss observed in our study was significantly higher than previously reported by Beg et al. in a study that examined *PTEN* deletions strictly in TNBC [19]. However, the findings of Lebok et al., who analyzed a large cohort of breast cancer patients, are in agreement with our results. They detected *PTEN* deletions in 43% of ER-negative tumors and *PTEN* gene loss was significantly correlated with ER/PR negativity [28].

Importantly, decreased PTEN protein expression significantly correlated with *PTEN* deletions in our study. Namely, all samples that had *PTEN* gene loss exhibited reduced or absent PTEN immunostaining (protein expression). Therefore, we concluded that deletion of the *PTEN* gene is a major genetic mechanism underlying the loss of PTEN protein expression in TNBC patients. However, 13% of samples with normal *PTEN* copy number showed loss of PTEN immunoexpression. Since *PTEN*-inactivating mutations are very rare in TNBC (according to The Cancer Genome Atlas Group, only one out of 93 TNBC/Basal samples harbored *PTEN* mutation) [29], it is likely that epigenetic mechanisms are responsible for PTEN inactivation in cases where the gene is not lost. For example, mir-21, which targets PTEN, is highly expressed in TNBC tissues [30].

Substantial disagreements exist regarding the effects of PTEN loss on TNBC clinical behavior. Some investigations found no connection between the loss of PTEN expression and clinical parameters of TNBC [16,17], while others indicated an association with lymph node involvement, disease-free survival, or OS [18,19]. We determined that homozygous *PTEN* deletions were associated with metastatic spread of TNBC, suggesting that tumors with complete PTEN loss were more aggressive. In support of this conclusion is the finding that PTEN suppresses metastatic dissemination [31].

There is uncertainty in the literature whether partial loss of PTEN is sufficient to enhance BC progression [28,32,33]. Our results support the complete inactivation model since homozygous deletions showed a dramatic effect on the DFI and OS of TNBC patients in univariate analysis, while there was almost no difference between hemizygous deletions and normal *PTEN* copy number in regard to patient survival. Therefore, our findings suggest a strong effect of homozygous *PTEN* deletions on TNBC prognosis. Consequently, TNBCs with a higher percentage of PTEN-null cells have a predisposition for metastatic dissemination and worse prognosis.

PTEN acts as a counterbalance to PI3K, which stimulates the PPAM survival pathway. PI3K is commonly activated in breast cancer by *PIK3CA* mutations. However, *PIK3CA* mutations are frequently found in hormone receptors (35%) and HER-2-positive tumors (23%), but are much less frequent in TNBC (8%) [34]. It seems as though PTEN loss is more prevalent in TNBC compared to *PIK3CA* mutations [35]. In addition, aberrations of mTOR are very rare [20]. Therefore, we decided to analyze the protein expressions of PI3K and mTOR. We determined that both PI3K and mTOR were highly expressed in TNBC (40% and 44%, respectively), with only 3% of cases showing negative PI3K or mTOR immunoreactivity (IHC score < 2). We showed that high PI3K expression was associated with larger size, lymphatic metastasis, and advanced tumor stage. Other studies support our findings and demonstrate an association of PI3K expression with pathohistological factors and unfavorable outcomes in various solid tumors [36,37,38,39]. High expression of mTOR in our cohort was also associated with advanced tumor stage but not with any other analyzed parameter. This is in line with previous studies, which found that mTOR expression in TNBC was mostly independent of clinicopathological parameters [40,41].

Furthermore, we detected a significant correlation in the expressions of PI3K and mTOR. Namely, TNBCs that had highly expressed PI3K also had a high level of mTOR protein expression. Moreover, when we examined PTEN expression in combination with PI3K and mTOR protein expression profiles, we observed significant differences between PTEN-reduced/PI3K-high/mTOR-high and PTEN-high/PI3K-low/mTOR-low groups in regard to pN stage, pathological prognostic stage, metastatic spread, DFI, and OS. Although multivariate analysis failed to reveal an independent prognostic value of the PTEN-reduced/PI3K-high/mTOR-high expression profile, the association with several adverse histopathological parameters and with poor DFI and OS in the univariate analysis strongly suggests that the PTEN-reduced/PI3K-high/mTOR-high profile might be a good prognostic factor and could be considered as a ‘high risk’ profile. A clinical trial of the AKT inhibitor ipatasertib demonstrated the importance of careful patient selection since the treatment effectiveness was higher in the *PIK3CA* mut/*AKT1* mut/*PTEN*-altered cohort [42]. However, *PIK3CA*/*AKT1* mutations are relatively rare in TNBC, while, as we have shown, PI3K and mTOR are commonly highly expressed in TNBC. The PTEN-high/PI3K-low/mTOR-low expression profile may allow identification of additional TNBC patients who could benefit from treatment with PPAM pathway inhibitors.

PPAM pathway activation enhances tumor aggressiveness and survival, which obviously also promotes TNBC chemoresistance. Our results indicated that this effect was independent from ABCB1, ABCC1, and ABCG2 expression. Though we found no significant connection between PPAM pathway activation and the expression of examined ABC transporters, both mechanisms were associated with TNBC and related to tumor progression and outcome [8]. Our findings support the combinatorial approach to TNBC treatment, using PPAM inhibitors together with ABC transporter modulators.

## 5. Conclusions

In conclusion, the loss of PTEN and high expressions of PI3K and mTOR are associated with poor outcome of TNBC patients. Our results imply that *PTEN* deletions constitute a major cause of reduced or absent PTEN expression in TNBC. Homozygous deletions of *PTEN* are potential molecular markers of metastasis formation in TNBC and might serve as robust predictors of TNBC outcome. The collaborative examination of PTEN/PI3K/mTOR protein expressions may be more useful in predicting TNBC clinical course than the expression of any single protein. Namely, PTEN-reduced/PI3K-high/mTOR-high profile has an important role in TNBC progression and is connected to unfavorable clinical course and outcome. Our results support the value of the PPAM pathway as a target for future TNBC anticancer therapies. Contrary to recent findings, we did not show significant connection between PPAM pathway activation and the overexpression of ABC transporters and, therefore, cannot confirm the thesis that PPAM directly promotes the MDR phenotype in TNBC patients. We strongly believe that these are two separated mechanisms that contribute to TNBC progression and unfavorable outcome; consequently, we believe that they should be considered as potential prognostic markers.

## Figures and Tables

**Table 1:** TNBC patients’ characteristics.

Parameters	np (%)
**Age at diagnosis**	
<50	19 (27)
≥50	51 (73)
**Tumor type**	
Ductal	41 (58)
Lobular	13 (18)
Other *	16 (24)
**Lymphovascular/perineural invasion**	
Absent	54 (77)
Present	14 (20)
Unknown	2 (3)
**Pathological prognostic stage**	
I	10 (14)
II	37 (53)
III and IV	23 (33)
**pT stage**	
T1	18 (26)
T2	43 (61)
T3 and T4	9 (13)
**pN stage**	
N0	36 (52)
N1	17 (24)
N2 and N3	17 (24)
**Histologic grade**	
I and II	45 (64)
III	25 (36)
**Metastases**	
M0	55 (79)
M1	15 (21)

Abbreviations: np, number of patients per group; *—medullary, tubular, and other rare carcinoma types.

**Table 2:** Association between gene deletions and protein expression of PTEN.

PTEN Reduced Expression	*PTEN* Gene Deletions			
	Yes	No	*p* Value	Kappa Value
**Yes**	31	9	<0.0001	0.75
**No**	0	30		

**Table 3:** Association between PTEN copy number/PTEN immunoexpression and clinicopathological parameters of TNBC.

Parameters	*PTEN* Copy Number Status				PTEN Expression		
	np (%)				np (%)		
	Wt	Hemi	Homo	*p* Value	Positive	Reduced	*p* Value
Patients	39 (56)	26 (37)	5 (7)		30 (43)	40 (57)	
**Age at diagnosis**							
<50	11 (28)	5 (19)	3 (60)	p^A^ 0.99	7 (23)	12 (30)	0.59
≥50	28 (72)	21 (81)	2 (40)	p^B^ 0.31	23 (77)	28 (70)	
				p^C^ 0.4			
**Tumor type**							
Ductal	23 (59)	15 (58)	3 (60)	p^A^ 0.06	19 (63)	22 (55)	0.07
Lobular	4 (10)	7 (27)	2 (40)	p^B^ 0.11	2 (7)	11 (27)	
Other *	12 (31)	4 (15)	0 (0)	p^C^ 0.13	9 (30)	7 (18)	
**Invasion** ^#^							
Absent	30 (81)	22 (85)	2 (40)	p^A^ 0.77	25 (86)	29 (74)	0.36
Present	7 (19)	4 (15)	3 (60)	p^B^ 0.08	4 (14)	10 (26)	
				p^C^ > 0.99			
**pp stage**							
I	8 (21)	2 (8)	0 (0)	p^A^ 0.24	6 (20)	4 (10)	0.49
II	18 (46)	16 (61)	2 (40)	p^B^ 0.38	15 (50)	22 (55)	
III and IV	13 (33)	8 (31)	3 (60)	p^C^ 0.31	9 (30)	14 (35)	
**pT stage**							
T1	11 (28)	7 (27)	0 (0)	p^A^ 0.98	9 (30)	9 (23)	0.69
T2	23 (59)	16 (61)	4 (80)	p^B^ 0.39	18 (57)	25 (62)	
T3 and T4	5 (13)	3 (12)	1 (20)	p^C^ 0.97	3 (13)	6 (15)	
**pN stage**							
N0	24 (62)	11 (42)	1 (20)	p^A^ 0.14	19 (63)	17 (42)	0.21
N1	6 (15)	9 (35)	2 (40)	p^B^ 0.19	5 (17)	12 (30)	
N2 and N3	9 (23)	6 (23)	2 (40)	p^C^ 0.17	6 (20)	11 (28)	
**Histologic grade**							
I and II	21 (54)	20 (77)	4 (80)	p^A^ **0.05**	16 (53)	29 (72)	0.13
III	18 (46)	6 (23)	1 (20)	p^B^ 0.37	14 (47)	11 (28)	
				p^C^ 0.07			
**Metastases**							
M0	35 (85)	21 (81)	1 (20)	p^A^ 0.24	26 (87)	29 (72)	0.24
M1	6 (15)	5 (19)	4 (80)	p^B^ **0.007**	4 (13)	11 (28)	
				p^C^ 0.74			

Abbreviations: np, number of patients per group; Wt, wild-type; Hemi, hemizygous deletion; Homo, homozygous deletion; pp stage, pathological prognostic stage; *—medullary, tubular, and other rare carcinoma types; #—data not available for two patients; p^A^—statistical significance between Wt *PTEN* and *PTEN* deletions (hemi- and homozygous); p^B^—statistical significance between Wt *PTEN* and homozygous deletions; p^C^—statistical significance between Wt *PTEN* and hemizygous deletions. Bold indicates statistically significant values, *p* ≤ 0.05.

**Table 4:** Association between PI3K/mTOR immunoexpression and clinicopathological parameters of TNBC.

Parameters	PI3K Expression			mTOR Expression		
	np (%)			np (%)		
	Low	High	*p* Value	Low	High	*p* Value
Patients	42 (60)	28 (40)		39 (56)	31 (44)	
**Age at diagnosis**						
<50	12 (29)	7 (25)	0.79	10 (26)	9 (29)	0.79
≥50	30 (71)	21 (75)		29 (74)	22 (71)	
**Tumor type**						
Ductal	27 (64)	14 (50)	0.48	24 (62)	17 (55)	0.54
Lobular	7 (17)	6 (21)		8 (20)	5 (16)	
Other *	8 (19)	8 (29)		7 (18)	9 (29)	
**Invasion** ^#^						
Absent	33 (80)	21 (78)	>0.99	31 (82)	23 (77)	0.76
Present	8 (20)	6 (22)		7 (18)	7 (23)	
**pp stage**						
I	10 (24)	0 (0)	**0.02**	9 (23)	1 (3)	**0.02**
II	20 (48)	17 (61)		21 (54)	16 (52)	
III and IV	12 (28)	11 (39)		9 (23)	14 (45)	
**pT stage**						
T1	14 (33)	4 (14)	**0.05**	13 (33)	5 (16)	0.14
T2	21 (50)	22 (79)		23 (59)	20 (65)	
T3 and T4	7 (17)	2 (7)		3 (8)	6 (19)	
**pN stage**						
N0	26 (62)	10 (36)	**0.04**	24 (62)	12 (39)	0.15
N1	6 (14)	11 (39)		7 (18)	10 (32)	
N2 and N3	10 (24)	7 (25)		8 (20)	9 (29)	
**Histologic grade**						
I and II	30 (71)	15 (54)	0.14	27 (69)	18 (58)	0.45
III	12 (29)	13 (46)		12 (31)	13 (42)	
**Metastases**						
M0	36 (86)	19 (68)	0.13	33 (85)	22 (71)	0.24
M1	6 (14)	9 (32)		6 (15)	9 (29)	

Abbreviations: np, number of patients per group; *—medullary, tubular and other rare carcinoma types, ^#^—data not available for two patients. Bold indicates statistically significant values, *p* ≤ 0.05.

**Table 5:** Association between PTEN/PI3K/mTOR coexpression and clinicopathological parameters of TNBC.

Parameters	PTEN -/PI3K+/mTOR+	PTEN +/PI3K-/mTOR-	*p* Value
	np (%)	np (%)	
**Age at diagnosis**			
<50	4 (31)	4 (28)	0.99
≥50	9 (69)	10 (72)	
**Tumor type**			
Ductal	5 (38)	8 (56)	0.28
Lobular	4 (31)	1 (8)	
Other *	4 (31)	5 (36)	
**Invasion**			
Absent	9 (69)	12 (84)	0.38
Present	4 (31)	2 (16)	
**pp stage**			
I	0 (0)	5 (36)	**0.01**
II	7 (54)	8 (57)	
III and IV	6 (46)	1 (7)	
**pT stage**			
T1	2 (15)	5 (36)	0.19
T2	9 (70)	9 (64)	
T3 and T4	2 (15)	0 (0)	
**pN stage**			
N0	4 (31)	12 (84)	**0.01**
N1	5 (38)	1 (8)	
N2 and N3	4 (31)	1 (8)	
**Histologic grade**			
I and II	7 (54)	10 (72)	0.44
III	6 (46)	4 (28)	
**Metastases**			
M0	7 (54)	13 (93)	**0.03**
M1	6 (46)	1 (7)	

Abbreviations: np, number of patients per group; *—medullary, tubular, and other rare carcinoma types. Bold indicates statistically significant values, *p* ≤ 0.05.

## Data Availability

The data presented in this study are available on request from the corresponding author.

## Supplementary Materials

The following are available online at https://www.mdpi.com/article/10.3390/life11111247/s1, Figure S1: Kaplan–Meier survival curves according to PI3K and mTOR protein expression in the TNBC group. DFI, disease-free interval, Table S1: Univariate analysis of tumor parameters as prognostic factors in patients with TNBC, Table S2: Multivariate model DFI, Table S3: Multivariate model OS, Table S4: PTEN, PI3K, mTOR protein expression correlation with the expression of ABCG2, ABCC1, and ABCB1 transporters.
