# Supplementary material for: Role of PTEN, PI3K, and mTOR in Triple-Negative Breast Cancer
Source: Life (Basel). 2021 Nov 17;11(11):1247. doi: 10.3390/life11111247 (PMC8621563; doi:10.3390/life11111247)
Supplement: Supplementary file 1 [file life-11-01247-s001.zip › life-1413054-supplementary.pdf]

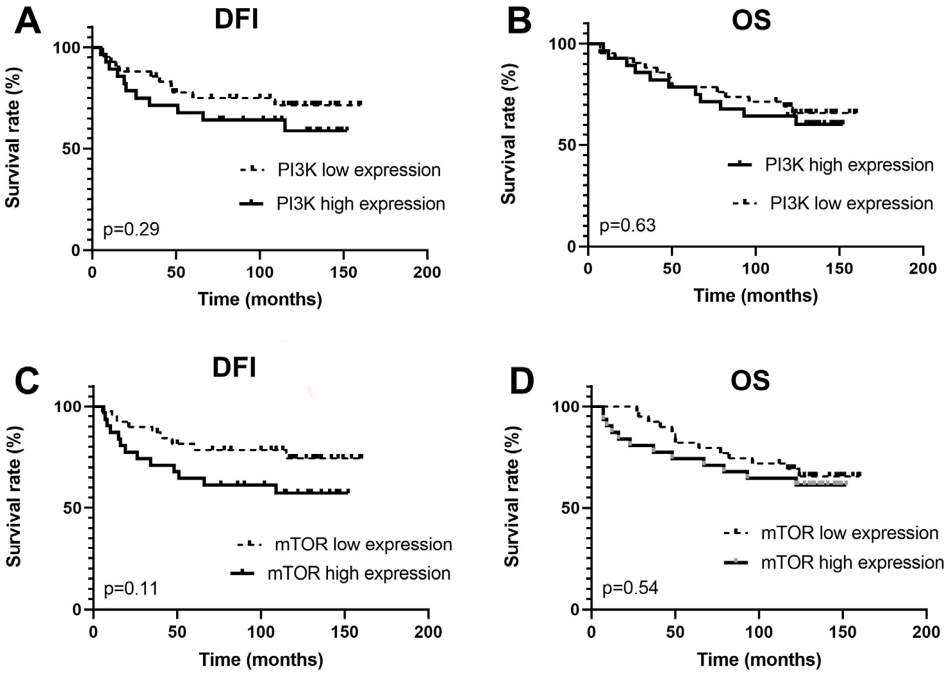

Figure S1. Kaplan–Meier survival curves according to PI3K and mTOR protein expression in the TNBC group. DFI, disease free interval; OS, overall survival; **A** PI3K expression did not affect patient DFI **B**. PI3K expression did not affect patient OS **C.** mTOR expression did not affect patient DFI **D.** mTOR expression did not affect patient OS.

Table S1: Univariate analysis of tumor parameters as prognostic factors in patients with TNBC

Parameters	Np (%)	Median DFI time (months)	p value	Median OS time (months)	p value
Number of patients per group	70 (100)				
Age at diagnosis					
<50	19 (27)	121	0.53	130	0.67
≥50	51 (73)	109		123	
Tumor type					
Ductal	41 (58)	98	0.33	124	0.54
Lobular	13 (18)	100		133	
Other*	16 (24)	118		120	
Lymphovascular/ perineural invasion					
Absent	54 (77)	115	0.001	130	0.005
Present	14 (20)	50		73	
Pathological prognostic stage					
I	10 (14)	108	0.52	125	0.08
II	37 (53)	109		127	
III and IV	23 (33)	109		122	
pN stage					
N0	36 (52)	116	0.01	130	0.002
N1	17 (24)	78		117	
N2 and N3	17 (24)	38		67	
pT stage					
T1	18 (26)	76	0.57	119	0.52
T2	43 (61)	110		131	
T3 and T4	9 (13)	114		130	
Histologic grade					
I and II	52 (67)	100	0.91	130	0.78
III	26 (33)	119		124	
Metastases					
M0	55 (79)	117	<0.0001	132	<0.0001
M1	15 (21)	20		41	

Table S2. Multivariate model DFI

Variables in the Equation

	B	SE	Wald	df	Sig.	Exp(B)	95.0% CI for Exp(B)	
							Lower	Upper
Lymphovascular/ perineural invasion	-.177	1.136	.024	1	.876	.838	.090	7.758
pN	-.022	.509	.002	1	.966	.978	.361	2.651
Metastasis	2.749	1.093	6.333	1	.012	15.634	1.837	133.074
PTEN_copy_number	-.061	.914	.004	1	.947	.941	.157	5.642
PTEN_PI3K_mTOR	1.179	1.589	.551	1	.458	3.253	.144	73.295

Table S3. Multivariate model OS

Variables in the Equation

	B	SE	Wald	df	Sig.	Exp(B)	95.0% CI for Exp(B)	
							Lower	Upper
PTEN_copy_number	.076	.315	.058	1	.809	1.079	.582	2.000
Lymphovascular/ perineural invasion	-.440	.505	.759	1	.384	.644	.239	1.733
pN	.203	.255	.633	1	.426	1.225	.743	2.018
Metastasis	2.927	.610	23.041	1	.000	18.679	5.653	61.728

Table S4: PTEN, PI3K, mTOR protein expression correlation with the expression of ABCG2, ABCC1 and ABCB1 transporters

	**ABCG2**		**ABCC1**		**ABCB1**	
**PTEN**	P value	R value	P value	R value	P value	R value
Whole cohort	0.46	-0.09	0.31	-0.12	0.21	-0.16
Lymphatic metastasis present	0.16	-0.25	0.11	-0.28	0.59	-0.09
Distant metastasis present	0.95	0.02	0.45	-0.21	0.53	-0.17
**PI3K**						
Whole cohort	0.61	0.06	0.32	0.12	0.91	-0.01
Lymphatic metastasis present	0.58	-0.09	0.9	0.02	0.58	-0.1
Distant metastasis present	0.15	-0.39	0.34	-0.26	0.63	-0.14
**mTOR**						
Whole cohort	0.92	0.01	0.25	0.14	0.45	0.09
Lymphatic metastasis present	0.21	0.22	0.16	0.25	0.76	0.06
Distant metastasis present	0.39	0.23	0.87	-0.05	0.72	0.09
